# Darwin’s tales–A content analysis of how evolution is presented in children’s books

**DOI:** 10.1371/journal.pone.0269197

**Published:** 2022-07-13

**Authors:** Isabell K. Adler, Daniela Fiedler, Ute Harms

**Affiliations:** IPN—Leibniz Institute for Science and Mathematics Education, Kiel, Germany; Texas State University, UNITED STATES

## Abstract

In science, certain theories led to a paradigm shift in human being’s approach to explain nature, such as the theory of relativity, the quantum theory, and the theory of evolution. The latter explains the emergence of biodiversity on Earth and all living beings’ relatedness, including humans. Accordingly, evolutionary theory is a central part of scientific literacy. However, scholars have demonstrated that misconceptions emerging in childhood hinder learners from grasping evolutionary processes. Implementing evolution in early science education could enhance scientific ideas as a basis for subsequent learning at school. Currently, children’s literature that deals with evolution is increasing and may enable more children to encounter evolutionary theory before entering school. This explorative study aimed to analyze how children’s books about evolution approach explaining this complex topic to young children in terms of covered contents, underlying concepts and use of language. We conducted (1) a text-based qualitative content analysis of 31 children’s books in the categories of organismal context, evolutionary principles, and misconceptions, and (2) a computer-supported content analysis of 33 word labels concerning (a) scientific terms and (b) verbs expressing evolutionary change. Although evolution is a universal concept, children’s books seem to promote specific contexts such as animal and human evolution. Even though the principle of selection requires an understanding of complex interactions between individuals and environmental factors, this principle was more frequent than the principles variation and inheritance. Phylogenetic history was covered more often than basic evolutionary processes, and evolutionary change was mainly mentioned at the species level over long periods. Besides, most books conveyed misconceptions such as transformationist, teleological or anthropomorphic reasoning. Consequently, books covering evolution may bias children’s first ideas concerning this topic or introduce unscientific ideas. Based on our results, we propose implications for early evolution educators and education researchers.

## Introduction

Children’s books are a powerful learning medium for science. First, they are one of the most commonly used media in families and daycare facilities [1, 2]. Second, they not only contribute positively to children’s cognitive development [3]; they can also engage them in learning scientific topics [4] and promote early biological knowledge [5]. As such, books can be used to introduce children to the topic of evolution to promote their scientific thinking and reduce ideas that may hinder subsequent learning (e.g., [6–8]). Despite the ongoing research in science education, evolutionary biology is still one of the topics in which learners of all ages face difficulties in achieving appropriate scientific understanding [9–11]. The theory of evolution explains the origin of biodiversity and the relatedness of all forms of life [12] and is the central concept of the life sciences taught in school (e.g., [13–15]).

More recently, researchers started investigating to what extent children can build conceptual knowledge about evolution and they also started to develop materials and interventions to foster children’s understanding of evolution (e.g., [7, 16–18]). At the same time, there has been an increasing trend toward the publication of literature on evolution for young children since the beginning of the 21^st^ century (i.e., 23 English and German publications within the last five years compared to 11 publications in the 15 years before; personal literature search). However, unless they constitute schoolbooks or intervention materials, children’s books are rarely evaluated for their effectiveness or scientific appropriateness, and authors of children’s books covering scientific phenomena are not required to have specific qualifications. In the past, children’s books have been found to have substantial shortcomings in language, content, and illustrations [19, 20]. Thus, although books can support early learning, they can be a source of misinformation or misinterpretation, too [21]. To date, there is no evidence on how published children’s books approach the challenging topic of evolution. Therefore, in this explorative study, we aimed to analyze children’s books about evolution to address the following questions:

1. How do children’s books about evolution present and explain evolution in terms of (a) examples (i.e., organismal contexts and evolutionary contents), (b) evolutionary principles (i.e., variation, inheritance, and selection), and (c) threshold concepts (i.e., spatial and temporal scales, randomness, and probability)?
2. To what extent do children’s books about evolution use unscientific reasoning (i.e., teleological, anthropomorphic, and essentialist reasoning)?
3. How do children’s books use scientific language (i.e., scientific nouns and verbs)?

## Background

Evolution education is a much-debated issue. A large part of the world’s population does not accept the theory of evolution and some countries oppose it being taught [22]; moreover, it is a counterintuitive concept [23] and even people that accept the theory of evolution have difficulties grasping it [24]. Recently, researchers and educators started implementing the topic of evolution in early science education. In the following sections, we will give an overview of the aspects of evolution that are dealt with in science education, namely, aspects of organismal context, evolutionary principles, threshold concepts, and misconceptions about evolution relevant for our content analysis. Moreover, on the basis of previous research, we will examine what children know and how they can learn about aspects of evolution.

### Reasoning about evolutionary change

#### Organismal context

Evolution is a universal principle that applies to all biological kingdoms (animals, plants, fungi, and bacteria) and even viruses [25]. However, when learners are confronted with a specific example, they tend to overestimate the importance of the task features and have difficulties identifying the underlying evolutionary concepts. Thus, learners demonstrate differences in knowledge and in misconceptions about evolution depending on the kingdom of the example chosen [26–29]. Although young children know that plants and animals share specific biological characteristics, they show differences when asked to assign biological concepts to plants or animals [30, 31]. Those differences might be linked to children’s ″living things concept‶. Given that children classify living things primarily on the basis of observable, goal-directed movements, they do not initially consider plants to be alive [31]. However, discussing biological processes has been shown to help young children to identify plants as living things [32]. Even though recent studies on children’s ideas about the evolution of plants are scarce, research indicates differences in learners’ knowledge and misconceptions about evolutionary principles with respect to the kingdoms [28, 33]. For example, some learners assumed that plants did not evolve or reproduce sexually, that they lacked within-species variation completely or varied only due to environmental factors (for an overview see [33]).

#### Evolutionary principles, key concepts, and threshold concepts

Several concepts are necessary to sufficiently explain evolutionary change [34–36]. Overall, evolutionary events can be described by setting them in a two-dimensional framework, using (1) the three evolutionary principles of variation, inheritance, and selection [12, 37] and (2) abstract, non-biology-specific threshold concepts (i.e., spatial scales, temporal scales, randomness, and probability; Author). Combining these concepts provides the necessary alignment between different problem contexts (e.g., organismal context, trait class, trait gain/loss) and helps learners to focus on the underlying nature of problems with different surface features (e.g., trait polarity; [38]).

The above-mentioned *evolutionary principles* form the first dimension of the framework and can be subdivided into varying numbers of key concepts. Altogether, they explain how variations between individuals (i.e., key concepts: *origin of variation*, *individual variation* (in contrast to between-species variation), and *differential fitness*) and the inheritance of these variations (i.e., key concepts: *reproduction* and *inherited variation*) can change populations through the process of selection (i.e., key concepts: *selection pressure*, *differential survival and reproduction rates*, *changes within populations*, and *speciation*; [39]).

Preschool children generally have low acceptance of within-species variation [40]. When they encounter the concept of variation through material that uses generic language or emphasizes a trait’s benefit, this can lead them to express more essentialist beliefs [41] (see also the following section: Misconceptions). However, it has been shown that these beliefs are susceptible to interventions [41]. Research on learning progressions described that children or novices notice differences in individuals in their daily lives and know that related organisms have different attributes even though those organisms have most traits in common [42, 43].

Concerning the principle of inheritance, young children seem to attribute more importance to kinship than to social relationships (like friendship) [44]. They tend to believe that parentage has more influence on height than on weight [45] and do not distinguish between the determinacy of physical traits compared to behavioral traits [46]. Children or novice learners do not know why individuals of a species look alike but should learn that organisms reproduce and pass their traits on to their offspring [43, 47].

When confronted with an impossible scenario of speciation (i.e., a cat becoming a horse), by the age of four, children mostly reject this type of reasoning, even though they largely accept extreme cases of metamorphosis [48]. Children and novice learners usually view change as a phenomenon that takes place at the individual level, for example, through growth [6, 42, 43]. However, intervention studies found that, at the age of seven, children can build a coherent understanding of common ancestry, natural selection, and speciation, and can generalize natural selection across different species and trait classes (e.g., [8]). A similar intervention with second graders also showed a decrease in different kinds of misconceptions [49]. In contrast, preschool children had more difficulties learning about natural selection and, instead, retained isolated facts [6, 7, 50–52]. Such difficulties may arise because it is more challenging for them to conceptualize abstract phenomena. However, it appears that a lack of coherent explanations and factual knowledge is often the limiting factor regarding children’s reasoning about aspects of evolution [7, 48, 49]. In addition, to date, no studies have conducted long-term interventions with preschool children [9].

The second dimension of the framework mentioned above is based on *threshold concepts* (e.g., [39]) that are connected to the evolutionary principles and key concepts. Threshold concepts assess different *spatial dimensions* (i.e., hierarchies that exist in biological systems, ranging from microscopic levels such as DNA or genes to macroscopic levels such as populations or species) and *time spans* (i.e., from extremely short scales such as mutations that occur in milliseconds up to deep time aspects such as cladogenesis that takes place over millions of years) in which evolutionary change can take place [39]. Furthermore, the evolutionary principles are influenced by stochastic and probabilistic events (concepts: *randomness* and *probability*; [53]). Understanding threshold concepts changes a learner’s view and can lead towards a more coherent and more in-depth understanding of the process of evolution (e.g., [39, 54]). For example, although learners can describe that mutations lead to variation, a deeper understanding of the concept of origin of variation can only be achieved when the concept of randomness is considered (i.e., variation is not a response of an individual to environmental changes but rather a prerequisite for natural selection to occur). Randomness here is defined as the lack of pattern or predictability of events (Author) and is mostly connected to the key concepts of origin of variation (e.g., randomness of mutations) or differential survival and reproduction rates (e.g., accidental deaths and random mating; [39]). A concept that is closely associated with randomness is the probability concept. Probability describes the likeliness or distribution of events that can occur and is particularly relevant for the key concepts of differential fitness (e.g., traits that influence chances of survival and reproduction) and differential survival and reproduction rates (e.g., environmental factors that influence chances of survival and reproduction; Author).

Time is an abstract concept and understanding large numbers and proportions is necessary to reason about large time scales [55]. Thus, children and even college students have only a flawed concept of large time scales [56–58]. This is especially relevant for the selection principle, which encompasses macroevolutionary processes, such as speciation, that span long periods of time. However, young children understand that living things have a past and a future [59] and, even though they do not have an elaborated concept of numbers, children often try to express that something lies very far in the past by using unrealistically large or imaginative numbers [60].

Concerning randomness and probability, previous research has shown that children depend primarily on visual data when estimating the outcome of events [61] and mostly deny the independence of consecutive events in random settings [62]. However, research has also shown that children’s probabilistic reasoning competence (i.e., understanding the connection between randomness and likelihood) is highly dependent on the domain and task [63]. Unfortunately, to the best of our knowledge, there has not yet been any research about children’s ability to understand randomness and probability in biological contexts.

#### Misconceptions

Within this study, we refer to *misconceptions* as conceptions or reasoning that contradict scientific knowledge [64]. Every person holds their own sets of ideas about scientific topics. However, possibly due to common cognitive biases, learners show similar beliefs about evolution, even across different ages and topics [64, 65]. The assessment of those beliefs can help researchers and educators to understand learners’ difficulties and to improve teaching strategies [64]. In the following, we describe the most common misconceptions about evolutionary biology that arise from teleological, anthropocentric, and essentialist thinking.

*Teleological reasoning* describes the idea that evolutionary change follows goal-directed causality [64], in which the function of a trait is interpreted as the reason for its existence, as is the case for human-made artifacts [11]. However, for evolutionary events, this reasoning pattern ignores the impact of variation, randomness, and probability [66].

*Anthropomorphisms* usually refer to the personification of physiological processes and the attribution of human characteristics to other organisms [67]. They are part of anthropocentric thinking, which also includes the overestimation of humans’ importance for the biological world [64]. In evolution, anthropomorphic thinking often results in the idea that organisms make conscious decisions to evolve [67].

*Essentialism* describes the idea that individuals of a population or species have static and uniform traits due to an underlying essence that defines their identity. It ignores, as does teleological reasoning, the impact of variation [64, 68]. Essentialists understand populations or species to be homogeneous groups that change at the same time and level through the act of inheritance and selection. In contrast, *transformationists* describe evolution as a transformation of individuals, populations, or species and do not consider the necessity of any underlying mechanisms [69].

All of the above-mentioned misconceptions have in common that they are often used as shortcuts by teachers, researchers, and science communicators to avoid long explanations or to entertain their learners [70–72]. However, instead of conveying scientifically adequate ideas, they cause or reinforce erroneous conceptions in learners [71]. For example, teachers tend to use anthropomorphic language to engage children’s interest and connect their experience and language with scientific knowledge. However, in biology lessons at a preschool, children seldom initiated anthropomorphic talk in scientific contexts themselves and sometimes even rejected anthropomorphisms [73].

In the context of selection, children especially show transformational and teleological reasoning or confound ontogeny with evolution [6, 49]. When explanations use evolutionary or need-based (i.e., teleological) reasoning, children show a higher level of selection understanding, while explanations that use desire-based (i.e., anthropomorphistic) reasoning have been shown to have a negative effect [74].

#### Scientific language

The skill of understanding and using scientific terminology is an integral part of scientific literacy and enables learners to communicate effectively about scientific phenomena [75]. Biology, in general, has a greater focus on and variety of scientific terminology than other scientific disciplines [76]. However, research shows that novice learners can only master a small subset of scientific terms [75–77]. Even though children are considered to acquire new words faster than adults (e.g., [78]), children have difficulties learning scientific terms from children’s books due to their length, unfamiliarity, and specific and abstract nature [79]. While most words that children learn in their mother tongue refer to concrete things that are already familiar, scientific terms refer to new ideas and concepts [80]. The use of a high number of different scientific terms in biology lessons seems to have adverse effects on students’ performance [77]. Thus, the higher the number of unknown words, the more difficulties children might have in comprehending a text and this might give rise to scientifically incorrect ideas [75].

## Methods

### Sample

Children’s books were collected using an explorative search strategy (see [72]) to imitate the natural searching behavior of web users. Because web users usually do not show linear searching patterns but shift from external web searches to internal search engines [81], we used Google and Google Shopping in combination with searches on the resulting book stores’ webpages (e.g., amazon.co.uk, bookdepository.com, buecher.de). We searched for the terms *evolution* and *children* (and the German equivalents) and selected those books that met the following criteria: (1) published and purchasable, (2) title or summary announced that the book deals with aspects of evolution, (3) published in English or German, and (4) recommended minimum age ranged from zero to six years. The search was carried out between October 2019 and April 2020. We classified all books into non-fiction books (NFBs; i.e., explain aspects of scientific subjects) or storybooks (SBs; i.e., tell the story of one or more protagonists’ experiences; [4]). The final sample consisted of 31 books (i.e., 13 NFBs and 18 SBs). A list of all analyzed children’s books is available in S1 Table.

### Content analysis

Based on a qualitative content analysis, we examined the books for their text-based implicit and explicit presentation of evolutionary biological content and misconceptions (see next section: Coding Scheme). A qualitative content analysis is a mixed-method that takes into account how texts are perceived and processed by readers [82, 83]. Our research team included experienced, native German-speaking evolution education researchers, who publish in reputable peer-reviewed journals (e.g., [53]), as well as doctoral candidates and undergraduate students. Two researchers coded the data based on the following coding scheme.

#### Coding scheme

The categories of evolutionary content were deductively derived from the work of Bohlin and colleagues [72] but were extended and differentiated during piloting to fit the aim and sample of this study. The final form included 62 variables, which were coded dichotomously as present or absent (Table 1).

**Table 1:** List of variables assessed in the content analysis.

Organismal Context		Principles and key concepts	Threshold concepts	Misconceptions
(Unicellular organisms)	Fungi	(Variation)	(Spatial scales)	Transformationism
				Teleology
• Unspecified	• Number of examples	• Origin of variation	• Molecule	Essentialism
			• Individual	
• Unicellular eukaryotes			• Population	Anthropomorphism
	• One species	• Individual variation	• Species	Evolution in waves
**Bacteria**	• Several species			Recent ancestry
		• Differential fitness		
• Number of examples	• Phylogenetic lineage		**(Temporal scale)**	
			• Seconds / minutes / hours / days	
• One species	• Real species	**(Inheritance)**	• Years	
• Several species	• Realistic species	• Reproduction	• Generations	
• Phylogenetic lineage		• Inherited variation	• Geologic timescale	
	• Fictitious species			
		**(Selection)**	• Time in numbers	
Real species				
• Realistic species	**Plants**	• Limited resources		
• Fictitious species	• Number of examples	• Differences in survival and reproduction rates		
			**Randomness**	
	• One species		**Probability**	
	• Several species	• Change in population		
**Animals**	• Phylogenetic lineage			
• Number of examples		• Speciation		
	• Real species			
	• Realistic species			
• One species				
	• Fictitious species			
• Several species				
• Phylogenetic lineage				
	**Symbols**			
• Real species				
• Realistic species				
• Fictitious species				
			
**Humans**				

Headings That Did Not Count as a Separate Variable in Parentheses

The category system and the coding manual are available in S2 Table.

*Organismal context*. We divided the organismal context (O; see S2 Table) into (1) general context (i.e., storyline) and (2) the context in which evolution was explained (i.e., explanatory parts). For the general context, we rated which kingdoms were mentioned within the whole book and counted the number of examples per kingdom (i.e., a reference to a species, class, or identifiable group of living organisms such as *vertebrates*, *reptiles*, or *Taraxacum officinale*, more specific than the generic term *animal* or *plant*; [84]). Concerning the evolutionary context, we rated (a) which evolutionary content (i.e., *evolutionary processes* [e.g., natural selection, isolation] or *phylogenetic lineages*) was used, (b) which kingdoms were used to explain evolution (*unicellular organisms [unspecified*, *eukaryotes*, *bacteria]*, *animals [including humans]*, *fungi* and *plants*), and (c) whether the examples were *real* (i.e., could be assigned to a species or genus without a doubt), *realistic* (i.e., were based on a real species, but could not be assigned to a species or genus without a doubt), or *fictitious* (i.e., were made up and not based on any real species).

*Evolutionary principles*, *key concepts*, *and threshold concepts*. For every book, we assessed whether the nine key concepts mentioned above (P; see S2 Table) were present or absent and on which spatial and temporal scales evolutionary events were presented (TC). Furthermore, we determined whether the concepts of randomness or probability were included. To determine the presence of evolutionary principles, we used and compared two different approaches [72]: In the first approach, only one associated key concept needed to be found in the books for the principle to be counted as present (relaxed approach). This served as an indicator of the principle’s general presence in the books. In the second approach, all associated key concepts had to be present (strict approach). This approach was used to determine the extent of complete representation in the books.

*Misconceptions*. We defined misconceptions (M; see S2 Table) as explicit errors or implicit, misleading explanations (i.e., reasoning not consistent with scientific ideas; [4]) such as *transformational* explanations (i.e., transformation of individuals or species is explained without mentioning any underlying mechanisms; see [6]), *teleological* explanations (i.e., traits evolve following a purpose; see [11]), *essentialist* explanations (i.e., evolution synchronously changes the inherent nature of all species members), *anthropomorphic* explanations (i.e., phenomena or living beings make conscious decisions to change), *evolution in waves* (i.e., evolution can start, stop, increase, or decrease), and *recent ancestry* (i.e., presentation of living species as ancestors).

#### Lexical search for scientific language

To analyze the scientific terminology, we conducted a computer-supported content analysis using the lexical search function in MAXQDA Analytics Pro 2018 (Release 18.2.3). The category system was created inductively during piloting and consisted of 33 word labels concerning (1) scientific evolutionary and biological terms (T1–T6) and (2) verbs to express evolutionary change (including nominalizations; T7–T15). Labels consisted of a key word, their German equivalent (e.g., *to become* and *werden*), and related (e.g., *ancestor* and *relative*) or synonymous words (e.g., *to become* and *to get*). We also examined in which context (i.e., evolutionary, geological, ontological, metabolic, non-biological, or conscious decision) the verbs appeared and whether a verb switched between the evolutionary meaning and another context (e.g., dinosaurs became birds vs. the girl became angry; fish grew lungs vs. humans grew food). Furthermore, we used the lexical search to support the analysis of organismal context (T16–21), evolutionary principles (T22–T24), and threshold concepts (T25–T33). A list of all word labels is available in S3 Table.

### Preparation of books and data analysis

To code the sample, we used the software MAXQDA and transferred the results to IBM SPSS Statistics 26 (Release 9.7.0.0.). To test reliability, we conducted an interrating analysis using one third of the material (*n* = 10; [82]). For each variable, we calculated (1) Krippendorff’s alpha using the SPSS KALPHA macro provided by Hayes and Krippendorff [85] using cut-off values of α_min_ = .67 for acceptable and α_min_ = .80 for reliable agreement [82] as well as (2) the percentage of agreement as a reference value in case Krippendorff’s alpha failed due to missing variation in the sample. The reliability for the complete coding scheme reached α_observed_ = .74. Disagreement was solved via discussion. An overview of all variables’ reliability scores is available in S4 Table.

For the lexical search, we extracted the text documents in MAXQDA, eliminated the title, book cover, and appendix, and proofread the text. We conducted a search for every label, as described in S3 Table, checked all hits for adequacy, and autocoded and transferred them to SPSS.

## Results

Based on the search criteria, 31 books were found (13 NFBs and 18 SBs), all published between 2003 and 2020. Twenty books were published in English and 11 in German. The NFBs contained between 23 and 63 pages, with seven to 167 words per page. The SBs spanned 13 to 42 pages, with six to 94 words per page. We only show a summary of our findings in this chapter (see Table 2). A more detailed description can be found in S1 File.

**Table 2:** Major findings of the content analysis for all categories in NFBs* (*n* = 13) and SBs (*n* = 18).

Category	Aspect	Variable	NFBs (%)	SBs (%)	Total (%)
Organismal Context	Kingdoms	Unspecified Cells	47	22	32
		Animals	92	100	97
		Humans	69	72	71
		Plants	62	61	61
	Evolutionary processes	Animals (incl. humans**)	46 (15)	50 (6)	48 (10)
		Plants	23	0	10
	Phylogenetic lineages	Animals (incl humans)	69 (69)	61 (44)	65 (55)
		Plants	8	6	7
Principles and Key Concept	Variation		54	56	55
		Origin of variation	15	11	13
		Individual variation	46	56	52
		Differential fitness	46	39	42
	Inheritance		62	67	65
		Reproduction	54	56	55
		Inherited variation	46	50	48
	Selection		77	89	84
		Limited resources	31	39	36
		Differences in survival and reproduction rates	54	56	55
		Change in population	31	28	29
		Speciation	62	56	58
Threshold Concepts	Spatial scales	Population	31	28	29
		Species	92	78	84
	Temporal scale	Years	69	61	65
		Time in numbers	69	56	61
	Randomness		15	17	16
	Probability		15	6	10
Misconceptions	Transformationism		85	67	74
	Teleology		54	50	52
	Essentialism		69	39	52
	Anthropomorphism		39	50	45
Lexical Analysis	Scientific terms		100 (Ø 44)	72 (Ø 12)	84 (Ø 25)
	Verbs of evolutionary change		92	89	90

* NFBs = non-fiction books; SBs = storybooks.

**Values refer to complete sample.

Overall, our findings show that specific contexts and contents tend to be preferred over others in children’s books about evolution. The analyzed children’s books named more and a greater range of different animal examples (*n* = 30; minimum: 1, maximum: 138) than plant examples (*n* = 18; minimum: 1, maximum: 16). To explain aspects of evolution, the books primarily used animals instead of plants and mainly focused on the context of phylogenetic history.

Selection was the most prominent and variation the least prominent evolutionary principle. Regarding the threshold concepts, most of the books treated evolution on the level of species and on a timescale of years. Almost all books contained misconceptions, with transformationism, teleology, and essentialism being the most prevalent. Most of the books used at least one scientific term and a verb to describe evolutionary change. The most frequently used verbs were *change* (*n* = 21; 68%) and *become/get* (*n* = 19; 61%). These verbs were often used across different contexts and often had different meanings within the same book.

## Discussion

In the following, we discuss our findings on how evolution is presented in the children’s books examined to gain insights into current strategies for explaining evolution to young children. Even though we discuss whether and, if so, how the analyzed aspects of evolution were addressed in the books, we do not consider it necessary that children’s books cover all aspects or that children need to understand every aspect of a book’s content. However, we agree that the content provided by educators should be selected purposefully to avoid frustration and negative attitudes towards science (e.g., [86, 87]). Thus, we conclude this article by presenting implications for children’s literature under the aspects of scientific accuracy and developmental appropriateness.

### Explaining evolutionary change in children’s books

#### Organismal context

Our findings indicate that children’s books about evolution favor animals and highlight their importance by using more and a greater range of different animal examples compared to plant examples. The strict focus on zoological content may favor an unbalanced distribution of knowledge and foster misconceptions about these two kingdoms. Even though plants are predominant in nature and are the driving power of our ecosystems (e.g., [88]), none of the books chose plants as protagonists or treated plant evolution in depth. This zoological bias, known as plant blindness (i.e., the inability to perceive plants in the environment and recognize their importance for humans and the biosphere; [89]), is part of the human perception, originates in infancy, and is also known to be a part of children’s environments and media [31, 90]. Knowledge of plant taxonomy and evolution could increase children’s awareness and appreciation of plants and the environment [91, 92]. However, the books we analyzed often did not even name the plants and instead referred to them solely as "plants", "flowers", "trees", or "forests" and, in seven cases, mentioned them only due to their function as habitat donors or food sources for animals. These findings are consistent with an analysis of educational videos in which Bohlin and colleagues [72] suggested that the preference for animals in evolution education may be due to familiarity and the similarities of animals with humans.

Concerning the number of examples used to explain aspects of evolutionary processes, all the NFBs mentioned more than one example, while the SBs referred exclusively to one example. One reason for this could be that the NFBs had more text per page. In addition, SBs, per definition, focus on a specific storyline, while NFBs cover a broader range of informative text. Moreover, the use of fictitious examples to explain evolution was found exclusively in SBs. Scholars in early evolution education often highlight the potential of fictitious examples because (1) fantasy creatures are not burdened with children’s prior knowledge [6, 41] and (2) children can transfer knowledge about a fictitious species to another fictitious [41] or real species [5]. However, due to the prevalent focus on Earth’s history, most of the books used a variety of real or realistic examples. Realistic examples occurred especially in books that mentioned reconstructed extinct ancestors (e.g., the first terrestrial animal). Using multiple examples and contexts might help learners to gain a deeper understanding of evolution by helping them to identify regularities and patterns through analogical encoding (i.e., learning by comparing between examples; [93]). However, using different examples to explain different phenomena could also lead to fragmented and context-dependent knowledge [38, 94].

Concerning the evolutionary context, the children’s books examined focused less on evolutionary processes and mainly on the evolutionary history of mammals. By doing so, they (1) often started with the origin of life, (2) presented eras with some characteristic extinct species, (3) mostly took human evolution as the endpoint, and (4) sometimes included depictions of phylogenetic trees without explaining how to read phylogenetic trees. These foci may—at best—be questionable due to the following reasons: First, biological evolution does not explain how life arose, but "only" explains the diversity and similarity of life on Earth [95]. Second, although the history of Earth and extinct species can be useful to illustrate processes such as speciation, relatedness, and extinction, describing different geological eras and understanding macroevolutionary processes requires a solid conception of evolutionary biology, deep time, randomness, and probabilistic processes to avoid a transformationist conception. Third, presenting evolution as a process in which less complex organisms become more complex ones can lead to the misinterpretation of evolution as a linear progression [56]. Lastly, phylogenetic trees can promote students’ understanding of evolution because phylogenetic trees visualize relationships among organisms or kingdoms (e.g., [56]) but they can also hinder learners’ understanding of variation and selection [96]. While older children mostly perform well after tree-reading interventions, preschool children face greater difficulties [51, 97]. As Earth’s history appears to be the most common way to teach evolution in children’s books, more research is needed that evaluates the potential of this topic and how it might be used best in early education [51].

#### Evolutionary principles and key concepts

The principles variation, inheritance, and selection are necessary to explain the process of evolution (e.g., [39, 98]). However, possibly due to the focus on Earth’s history rather than on general aspects of evolution, the examined books seemed to reduce evolution to the selection principle, thereby underestimating the value of understanding variation and inheritance. While variation and inheritance describe observable phenomena in individuals and their offspring, natural selection describes a highly complex interaction between the organisms of a population and environmental factors over long time spans. A recent intervention study about speciation showed that elementary school children were able to understand and generalize the logic of speciation after a unit about variation, inheritance, and selection had been taught [8]. However, the books examined that favored such a species perspective rarely covered variation and inheritance.

In contrast to selection, variation is a directly observable part of children’s everyday life. It can be observed in humans and, hence, can be projected easily onto other organisms [67]. However, biology learners often ignore the importance of within-species variation even though it is the precondition of selection [54, 99]. Provine [100] criticized that the common focus on selection and the ignoring of variation and inheritance could lead people to reject the theory of evolution. When evolution is reduced to the principle of selection, people not only develop misconceptions about evolution but also ignore other evolutionary mechanisms such as the increase in mutant variants and heterozygosity in the human gene pool [100, 101]. Our analysis revealed that this trend might begin early in evolution education, and it might be reinforced in further education when variation and inheritance do not receive sufficient emphasis in biology classes. However, more research on whether an early understanding of variation can favor the initiation of evolutionary knowledge is needed [9].

#### Threshold concepts

As the principle of selection and especially the key concept of speciation were predominantly favored in the books, it is not surprising that nearly all of the books showed evolutionary events on the scales of species, years, and absolute large numbers. Prioritizing a species perspective in children’s books and focusing on species as evolving units could potentially reinforce essentialist and teleological thinking as well as the underestimation of the role of random mutations, to which children are particularly prone when expressing their ideas about speciation [99, 102]. Possibly due to the abstract nature of microscopic structures, evolutionary events on a molecular scale were scarcely dealt with in the children’s books we examined. Previous research has shown that even students and adults show difficulties imagining DNA and understanding the connection between genes and morphological or physiological traits [103]. However, the implementation of a simplified gene concept in order to explain inheritance can help children to shape their reasoning and can reduce anthropomorphic ideas [104]. Thus, carefully inserted metaphors in books might help children to initiate preliminary ideas about biological concepts. As an example, one SB described DNA as a tiny book that contained all the information about a fictional creature’s properties [105]. Studies have shown that students inherently use metaphors when thinking about DNA (e.g., DNA is metaphorically described as a kind of data storage; [106]). Yet, further research is necessary to examine the effects of such metaphors when used in long-term interventions or interventions that involve active thinking about metaphors and their biological ideas.

Even though young children do not yet have a solid conception of absolute time, they order events by their occurrence and, at the age of five, already show a concept of relative time [60, 107]. Hence, they might be able to learn about the order of when distinct species lived on Earth. However, children at the age of five more easily understand metaphors about the temporal relationship between a person and an event than about the temporal relationship between two events from an implicit observer perspective [108]. Thus, it could be argued that it might be difficult for children to imagine Earth’s history as a narrative about a series of events from an implicit observer perspective.

Even though an understanding of the concepts of randomness and probability is proposed to reduce misconceptions [39], these concepts were nearly not touched on in the books examined. Due to natural selection, changes in populations often appear to be directed, and students tend to underestimate the role of randomness and to consider an internal or external force or need as the driving power of evolutionary change [109]. On the one hand, by neglecting to acknowledge the random nature of evolutionary events, children’s books may reinforce teleological misconceptions regarding the variation principle. On the other hand, even adults often lack a coherent understanding of the meaning of randomness and children might have even more difficulties [62]. Although some studies have shown that infants can reason about randomness and probability in the physical and psychological domain (e.g., [110]), there are no studies on the probabilistic reasoning of children in biological contexts.

#### Misconceptions

Nearly all of the books included representations that might foster misconceptions, while implicit misconceptions appeared more often than explicitly wrong information. The NFBs analyzed provided more transformational, teleological, and essentialist misconceptions, whereas the SBs contained more anthropomorphic misconceptions.

Transformational language appeared in most of the books due to fragmentary content. Especially the NFBs and SBs that treated phylogenetic lineages without covering explanations about evolutionary processes contained transformational reasoning more often than books that touched on evolutionary processes. One can argue that understanding evolution as the transformation of a species may be considered an initial idea toward a deeper understanding of evolution. However, in learning about evolutionary logic, children benefit from causally coherent explanations that use sufficient mechanistic specification to prevent explanatory gaps prone to unsupervised reinterpretation [6].

Teleological ideas are not often expressed by young children when they are asked about evolution [6] but teleological ideas seem to start impacting children’s reasoning at the end of preschool [111]. Teleological reasoning can help children to identify plants as living things [31]. However, teleological explanations have to be implemented carefully. For example, selection teleology is a valid explanation for why individuals of a species have certain traits (i.e., a trait is considered to occur because of its consequences, and a design stance or an adaptationist view is not inferred). However, all books that made use of teleological reasoning exclusively used internal and external design teleology, which are not valid explanations [11].

Essentialism was often implicitly expressed through generic language (i.e., the attribution of a trait to a whole species; [112]) in the books we examined. Children have the innate tendency to find underlying, or rather abstractable, characteristics [68]. Thus, even though generic language appears to be very subtle, children have been shown to be highly susceptible to interpreting information that uses generic language as being conceptually relevant (i.e., identity-defining; [112]).

Anthropomorphic explanations about evolution transfer a biological topic into a psychological context that underlies different patterns, such as intentionality. Anthropomorphisms can engage children in science and trigger their interest and preknowledge [73]. However, anthropomorphic language can hinder learning if it remains at the metaphorical level [73], which was the case for most of the books analyzed in our study. Anthropomorphisms can make stories more illustrative or entertaining by giving the protagonists the ability to induce evolution, to express traits, or to demonstrate speciation through conscious decision-making. Adults usually use anthropomorphisms to make children enjoy scientific explanations or when they lack knowledge themselves or underestimate children’s potential [73, 113]. However, it has been shown that children retain erroneous anthropomorphic explanations about evolution better than scientific explanations [74].

The underlying complexities of Earth’s history and humans’ phylogeny are difficult to explain within a few sentences and in a highly simplified manner. Misconceptions, therefore, may serve as shortcuts in books to avoid complex scientific explanations. Books can help both children and adults to understand a scientific topic and to engage in conversation. Yet, if a book does not provide meaningful explanations, readers need to provide additional explanations, and not all parents might be able to provide such explanations, due to a lack of willingness, knowledge, or educational expertise [67, 114]. Thus, books should—at least try to—include as few misconceptions as possible, even if this results in longer text elements.

Overall, it appears that children’s books on evolution show similar tendencies towards plant blindness, misconceptions, and poor transfer of the evolutionary logic between different levels as students do. One may attribute this to psychological biases that all humans share and that result in common misconceptions [23, 64, 69].

#### Scientific language

In the children’s books examined, we found a high usage of biology-specific terminology (i.e., evolutionary terms such as adaptation, selection, rudiments, and other biological terms such as population, cells, and DNA), which is probably not part of children’s daily interaction in most households. Even though children are considered to acquire new words faster than adults (e.g., [78]), children might have difficulties understanding unfamiliar, long, specific, and abstract scientific terms from children’s books [79]. While most words that children learn in their mother tongue refer to concrete things that are already familiar to them, scientific terms refer to new ideas and concepts [80]. Thus, the higher the number of unknown words, the more difficulties children might have in comprehending a text [75]. Even though SBs typically use everyday language [4], the SBs in our study also included scientific terms, but to a lower extent than the NFBs. Yet, educators usually engage more in extra textual talk and scaffolding when reading NFBs because they read selected parts only [115]. Hence, teachers might compensate for the higher number of scientific terms in NFBs.

To describe evolutionary change, nearly all of the books used active verbs (e.g., *evolve*, *adapt*, *change*). The reason for this was probably to reduce the length of explanations in the books but it led to simplistic explanations. Moreover, these verbs might lead to misinterpretations because explanations that contain active verbs appear to be fragmented or circular. Furthermore, they carry a complex, theory-embedded meaning that differs from their meaning in everyday language [79, 99, 103, 116]. For example, the verbs *evolve* and *adapt* were exclusively used in the evolutionary context, referring to the change in the frequencies of traits that turn out to be beneficial in a particular environment within a population over generations [99]. However, in everyday language, the verb *adapt* is used to express that a single organism adjusts itself to its surrounding conditions within its lifespan. Although students use this verb to describe evolutionary change, they often understand it in the sense of its everyday meaning [99]. The confusion of scientific meaning with everyday meaning may even be fostered by the fact that most books used the verbs in different contexts (e.g., the *development* of wings versus the *development* of tools). It is unlikely that children grasp the scientifically adequate meaning of such verbs if the verbs are not embedded in age-appropriate explanations or are not explicitly detached from the everyday meaning; in the books we analyzed, such explanations were mostly absent.

### Limitations

This article discussed the trends of how children’s books try to convey evolutionary content. The central weakness of this study is that our assumptions concerning the supporting or hindering effects of children’s books for learning evolution remain theoretical. The judgments we made were strongly dependent on the extent and quality of existing studies and they need further empirical testing. The interaction between book and recipient is complex [19] and, in this case, is even more complicated when children read literature with their parents or siblings, who provide additional information or misconceptions. Moreover, our analysis focused exclusively on text elements. While pictures and illustrations make the content appealing and memorable, they can also encourage conversations during reading activities between the reader and the listening child [4]. Thus, future studies could focus on analyzing how evolution is represented pictorially in children’s literature and how children might understand these representations. Concerning the text elements, it was sometimes difficult to differentiate between misconceptions and unprecise explanations, as reported by Mills Shaw et al. [103]. Unclear or tendentious phrases were mostly rated as misconceptions because (1) children lack the prior knowledge required to accurately interpret an insufficient explanation, and (2) even subtle language changes can significantly impact children’s interpretation and understanding of content [112]. As a consequence, the categories of misconceptions scored low in reliability. This effect was also reported by Bohlin and colleagues [72]. In addition, some categories scored low in reliability due to the lack of variation in our sample, so conclusions cannot be drawn about the quality of those categories.

### Implications for science education researchers

Early evolution education is a complex topic that needs to cope with different delicate features (e.g., specific contexts, misconceptions, or ambiguous wording) that can bias the first understanding of evolution and may occur in children’s books due to their reductionist nature. Early evolution education is a growing field and, in our study, we found some significant tendencies that should be considered in further education research. First, we found that the books nearly completely neglected plant evolution. As children perceive plants as living later in their development than when they perceive animals as living (e.g., [117]), future research should examine whether younger children show context-dependent misconceptions, as has been found in older students (e.g., [28]). Second, we found that evolutionary key concepts occurred in an unstructured way, and most of the books treated only selection and Earth’s history as central aspects of evolution, while variation and inheritance were widely ignored. In contrast to such an approach, we would like to argue that evolution education does not always necessarily have to cover selection and that classes about variation and inheritance can justifiably be seen as lessons about evolution. Further research should investigate whether the early integration of variation or inheritance can promote evolutionary ideas in children and reduce misconceptions. Third, in reviewing current literature, we found that there is much uncharted ground in early evolution education, especially regarding threshold concepts. For example, there is little research on the extent to which children can make sense of long time spans and on strategies that might help children to conceptualize not only the sequence of events but also the metrics of timelines. Although studies have explored probabilistic thinking in infants and children, there are no studies on children’s understanding of randomness and probability in the biological context. It would be intriguing to know whether children benefit from books or explanations about evolution that include simplified explanations or metaphors about randomness and probability. Fourth, misconceptions related to anthropomorphisms, teleology, and essentialism are a central issue in evolution education. However, we also found transformationism to be a ubiquitous misconception in children’s books. In connection with this, the verbs that described evolutionary change, as identified by our lexical search, were prevalent in almost all books, and we found that they were associated with incomplete explanations and transformational language. Future research could examine the role of transformational language in biology classes and textbooks. Lastly, although the publication of children’s literature about evolution has increased rapidly since the turn of the century, there are no assessments of current literature or recommendations based on empirical studies on how to ensure the scientific accuracy and developmental appropriateness of the contents. It seems that most publishers do not sufficiently ensure the content quality of children’s books. Thus, we hope that there will be further projects that involve researchers and provide scientifically flawless materials for early evolution learning [see 17, 18, 105, 118, 119].

### Implications for early childhood educators

Children’s books can be a gateway to initiating a more coherent and complete understanding of evolution. Based on our analysis, the following implications can be addressed to guide early formal and informal learning about evolution and to help educators in choosing valuable books for their young learners.

First, addressing plant life in children’s literature about evolution is important in order to tackle plant blindness. To foster taxonomic and evolutionary knowledge about plants, educators could examine whether a book includes plants and, if not, consider addressing the issue themselves and consider teaching children that plants are living things that differ in their phenotypes and reproduce, just as animals do.

Second, educators could assess whether a book includes variation and inheritance before introducing natural selection or they could preface a unit on these topics before beginning to read the book. Research suggests that the order in which elements of a topic or skill are introduced (i.e., from basic to more complex phenomena) may have a greater impact on learning than the age of the learners [120]. Further, they should consider waiting at least until elementary school to introduce the first key concepts of selection because it seems that it is easier for elementary school children to build a conceptual understanding of natural selection [50].

Third, children’s books should obviously not contain erroneous information or foster misconceptions. Children appear to be highly susceptible to ideas that fit into their intuitive mental models. Detecting misconceptions might not be easy as we found that the most common misconceptions were transformationisms, which are not scientifically incorrect per se, but leave explanatory gaps that are prone to children’s misinterpretations [6]. Educators should be aware of the fact that not all children’s books are written by professionals and they should intervene if a book contains incorrect or misleading information. This would require educators to acquire some knowledge of misconceptions and how to detect them. Again, variation and inheritance are low-key principles for early evolutionary teaching that do not require much prior knowledge. Their presence and the absence of scientific terms could also be a first indicator that selection may not be explained in terms of transformational, teleological, essentialist, or anthropomorphic language.

Fourth, scientific terminology can be a barrier to learning biology and should be chosen carefully [76]. Books should avoid shortening explanations by leaving out essential information and should avoid using ambiguous, abstract scientific terms. However, when educators find many scientific terms in a book, they could provide additional explanations for their young learners or replace those terms with more everyday expressions as long as their everyday meaning does not conflict with the scientific meaning [121]. Examples for this were found in some of the books (e.g., *group* instead of *population*; *surprise* instead of *randomness*). Kelemen and The Child Cognition Lab [17, 18] showed that it is possible to explain natural selection without using scientific terms or any of the verbs mentioned above that express evolutionary change.

Fifth, we recommend introducing the topic of evolution with an SB. SBs are more familiar to children and are particularly appropriate for young learners both with and without prior knowledge. SBs can engage children’s imagination and this might enable children to link their knowledge to the views of fictional characters and to approach problems from another person’s perspective [122]. Furthermore, the narrative texts of SBs, compared to the informative texts of NFBs, are usually more familiar to children and help them to grasp aspects of evolution as such texts fuel their imagination and facilitate the understanding of chronology, causes, and consequences [115, 122]. NFBs tend to contain more scientific terms and longer texts. However, they are rarely read completely in class and encourage children to interact and engage in conversation with the readers [115].

Finally, children’s books should not try to "fill a gap" by attempting to answer all of the questions that children may have on the topic. Science education is often text-oriented and aims at memorization [86]. However, children’s books and educators should aim to open up children’s intrinsic motivation to discover and interact with the biological world and to engage in conversations with peers or adults [20].

## Supporting information

S1 Table Children’s books about evolution that were included in the content analysis. (PDF)

S2 Table Category system used in the content analysis concerning organismal context (O), principles and key concepts (P), threshold concepts (TC), and misconceptions (M). (PDF)

S3 Table Dictionary of the computer-supported content analysis. (PDF)

S4 Table Results of the content analysis including reliability scores concerning organismal context (O), principles and key concepts (P), threshold concepts (TC), and misconceptions (M). (PDF)

S1 File Detailed results of the content analysis. (PDF)
