# Supplementary material for: Darwin’s tales–A content analysis of how evolution is presented in children’s books
Source: PLoS One. 2022 Jul 13;17(7):e0269197. doi: 10.1371/journal.pone.0269197 (PMC9278771; doi:10.1371/journal.pone.0269197)
Supplement: S1 Table — (PDF) [file pone.0269197.s001.pdf]

Supporting information

S1 Table

Children's Books About Evolution That Were Included in the Content Analysis

No.	Title	Author(s)	Publisher	Category	First pub.	Age recom. from
**1**	How worm became everything: The story of evolution	Darkin, C.	Rational Stories	SB	2019	0
**2**	Evolution für Babys [Evolution for babies]	Ferrie, C. & Florance, C.	Loewe	NFB	2019	2
**3**	The story of everything	Layton, N.	Barron's Educational Series Inc	NFB	2006	3
**4**	Grandmother fish: A child's first book of evolution	Tweet, J.	Feiwel and Friends	NFB	2015	3
**5**	Ich und der Anfang der Welt [How did I get here?]	Bunting, P.	Gabriel	NFB	2018	4
**6**	Warum hat die Giraffe einen langen Hals?: Wie Tiere sich ihrem Lebensraum anpassen [Why does the giraffe have a long neck? How animals adapt to their environment]	Kim, E. H.	Fischer	NFB	2008	4
**7**	The evolution of humans according to Uncle Charles	Professor Beaver	Professor Beaver	NFB	2017	4
**8**	Annabelle & Aiden: The story of life (an evolution story)	Becker, J. R.	Imaginarium Press, LLC	SB	2016	4
**9**	Oh, the things we believed!	Becker, J. R.	Imaginarium Press, LLC	SB	2017	4
**10**	How the Borks became	Emmett, J.	Otter-Barry Books	SB	2019	4
**11**	Galapagos George	George, J. C.	Harper Collins	SB	2014	4
**12**	Das große Tier [The big animal]	Jossen, P.	Moritz	SB	2017	4
**13**	One day a dot: The story of you, the universe, and everything	Lendler, I.	First Second	SB	2018	4
**14**	From lava to life	Morgan, J.	Dawn Publications	SB	2003	4
**15**	Mammals who morph	Morgan, J.	Dawn Publications	SB	2015	4
**16**	Charlie and the tortoise	Mouton, M. J.	Secular Media Group	SB	2016	4
**17**	Evolution: Science explained for our little ones	Pazos, C.	Sky Pony Press	SB	2020	4
**18**	Charlie and Kiwi: An evolutionary adventure	Reynolds, P. H. & The New York Hall of Science	Atheneum Books for Young Readers	SB	2011	4
**19**	Our family tree: An evolution story	Westberg Peters, L.	Harcourt	SB	2003	4
**20**	Ein kluger Fisch [One smart fish]	Wormell, C.	Beltz & Gelberg	SB	2010	4
**21**	It started with a big bang: The origin of life, you and everything else	Bal, F.& van Doninck, S.	Kids Can Press	NFB	2018	5
**22**	Island: A story of the Galápagos	Chin, J.	Scholastic Inc	NFB	2012	5
**23**	Darwins Entstehung der Arten [Charles Darwin’s On the Origin of Species]	Radeva, S.	Carl Hanser	NFB	2019	5
**24**	Faszinierende Evolution: Wie das Leben entstand [Amazing evolution: The journey of life]	Claybourne, A.	Kosmos	SB	2019	5

**25**	Die Welt sagte ja [The world said yes]	Dahle Nyhus, K.	Kullerkupp Kinderbuch Verlag	SB	2019	5
**26**	Die Welt der Höpfe: Eine kleine Geschichte über die Evolution [The world of the Hopfs: A small story about evolution]	Heuschele, J.	CreateSpace Independent Publishing Platform	SB	2015	5
**27**	Moth: An evolutionary story	Thomas, I. & Egnéus, D.	Bloomsbury Children's Books	SB	2018	5
**28**	The story of life: A first book about evolution	Barr, C.	Francis Lincoln Children's Books	NFB	2015	6
**29**	Evolution für Kinder [Evolution for children]	Eichinger, W.	Qantor Verlag	NFB	2011	6
**30**	Dieses Huhn ist ein T-Rex! Ein Buch über die Evolution der Tiere [This chicken is a T-Rex! The great big book of animal evolution]	Garcia Mora, R.	Edizioni White Star SrL	NFB	2017	6
**31**	Little changes: An introduction to evolution	Taylor, T.	CreateSpace Independent Publishing Platform	SB	2012	6
