# Supplementary material for: Darwin’s tales–A content analysis of how evolution is presented in children’s books
Source: PLoS One. 2022 Jul 13;17(7):e0269197. doi: 10.1371/journal.pone.0269197 (PMC9278771; doi:10.1371/journal.pone.0269197)
Supplement: S2 Table — (PDF) [file pone.0269197.s002.pdf]

Supporting information

S2 Table

Category System Used in the Content Analysis Concerning Organismal Context (O), Principles and Key Concepts (P), Threshold Concepts (TC), and Misconceptions (M)

Code	Variable	Operationalization	Example
O	**Organismal Context**		
**O1**	**Unicellular organisms**		
**O1_UN**	Unspecified	Does the book mention unspecified living cells?	"This first life was just an incredibly small shapeless blob, called a cell." ([1], counted* p. 4)
**O1_EU**	Unicellular eukaryotes	Does the book mention unicellular eukaryotes?	"Two battling bacteria tried to turn each other into a liquid lunch. Instead, they merged into a new Earthling: a Eukaryote." ([2], p. 19)
**O2**	**Bacteria**	Does the book mention members of the bacteria kingdom?	"That ancient living stew brought forth the very first Earthlings. They were Bacteria." ([2], p. 11)
O2_AM	*Number of examples*	How many examples are given per kingdom? An example is defined as a reference to a species, class, or identifiable group of living organisms, which is more specific than the generic term "animal" or "plant".	
O2_S1	*One species*	Is evolution shown in the context of one example?	
O2_S2	*Several species*	Is evolution shown in the context of more than one example?	see examples of O3
O2_PL	*Phylogenetic lineage*	Is evolution shown in the context of a phylogenetic lineage?	
O2_RL	*Real species*	Does the book use real examples to explain evolution?	
O2_RT	*Realistic species*	Does the book use realistic examples to explain evolution?	
O2_FK	*Fictitious species*	Does the book use fictitious examples to explain evolution?	
**O3**	**Animals**	Does the book mention members of the animal kingdom?	"We know that birds changed by looking at their ancestors, like this ancient fossil. " ([3], counted p. 17)
O3_AM	*Number of examples*	How many examples are given per kingdom?	"'Some birds don't fly,' explained Grandpa Charles. 'Kiwis, penguins, and ostriches.' (3 animal examples; [3], counted p. 16)
O3_S1	*One species*	Is evolution shown in the context of one example?	"How did the Borks become what they are now?" ([4], counted p. 6)

Code	Variable	Operationalization	Example
O3_S2	*Several species*	Is evolution shown in the context of more than one example?	"Some seagulls begin to hunt at night. In time their eyes become larger, allowing them to see better in the dark. The tortoises' shells change shape. As the land becomes drier, their shells become smaller and turn up in front." ([5], counted p. 14)
O3_PL	*Phylogenetic lineage*	Is evolution shown in the context of a phylogenetic lineage?	"'And over time, the birds evolved into thousands of kinds of birds!' 'Kiwi-you're a kind of dinosaur!'" ([3], counted p. 35)
O3_RL	*Real species*	Does the book use real examples to explain evolution?	"Die Giraffe hat einen langen Hals, weil sie ihre Nahrung auf Bäumen findet." ([6], counted p. 31) [Translated: The giraffe has a long neck because it finds its food on trees.]
O3_RT	*Realistic species*	Does the book use realistic examples to explain evolution?	"Aber der erstaunlichste Fisch von allen… war der da." ([7], counted pp. 7–10) [Translated: But the most amazing fish of all... was this one.]
O3_FK	*Fictitious species*	Does the book use fictitious examples to explain evolution?	"They were called the rinkidinks." ([8], p. 2)
**O3_1**	**Humans**	Does the book mention members of the genus Homo?	"Und seit einem Urahn, den wir uns mit den Affen teilen, entwickelten wir uns nach und nach zu dem, was wir heute sind. Menschen." ([9], p. 21) [Translated: And since an ancestor that we share with monkeys, we evolved little by little into what we are today. Humans.]
**O4**	**Fungi**	Does the book mention members of the fungi kingdom?	"They found each other and became the very first plants and fungi." ([2], p. 27)
O4_AM	*Number of examples*	How many examples are given per kingdom?	
O4_S1	*One species*	Is evolution shown in the context of one example?	
O4_S2	*Several species*	Is evolution shown in the context of more than one example?	
O4_PL	*Phylogenetic lineage*	Is evolution shown in the context of a phylogenetic lineage?	see examples of O3
O4_RL	*Real species*	Does the book use real examples to explain evolution?	
O4_RT	*Realistic species*	Does the book use realistic examples to explain evolution?	
O4_FK	*Fictitious species*	Does the book use fictitious examples to explain evolution?	
**O5**	**Plants**	Does the book mention members of the plant kingdom?	"Soggy weeds stretched towards the sun – very, very slowly evolving into tall trees." ([1], counted p. 10)
O5_AM	*Number of examples*	How many examples are given per kingdom?	
O5_S1	*One species*	Is evolution shown in the context of one example?	
O5_S2	*Several species*	Is evolution shown in the context of more than one example?	see examples of O3
O5_PL	*Phylogenetic lineage*	Is evolution shown in the context of a phylogenetic lineage?	
O5_RL	*Real species*	Does the book use real examples to explain evolution?	

Code	Variable	Operationalization	Example
O5_RT	*Realistic species*	Does the book use realistic examples to explain evolution?	
O5_FK	*Fictitious species*	Does the book use fictitious examples to explain evolution?	
**O6**	**Symbols**	Does the book use inanimate objects or shapes to illustrate evolution?	"Aber schau! Es wurde ein neuer Ball gemacht, der nicht durch das Loch passt. Und dieser Ball kann neue Bälle machen!" ([10], counted pp. 16–17)<br>[Translated: But look! A new ball was made, which does not fit through the hole. And this ball can make new balls!]
**P**	**Principles and Key Concepts**		
**P1**	**Variation**		
P1_ORI	Origin of variation	Does the book mention that within-species variation originates at the genetic level or at the level of inherent information that can determine physical aspects and/or can be inherited?	"Diese Unterschiede entstehen durch Mutationen und andere Kombinationen der Eltern-DNA." ([11], p. 17)<br>[Translated: These differences are caused by mutations and other combinations of the parents' DNA.]
P1_IND	Individual variation	Does the book mention that individuals in a population are not identical but vary in different characteristics?	"The thing about Borks is, no two are a match. They're all a bit different, just look at this batch." ([4], counted p. 3)
P1_FIT	Differential fitness	Does the book mention that individuals have different chances of survival and reproduction in their environment, due to their varied characteristics?	"But say one family was a little bit different. Say some stayed on the ground a little more and smelled bugs a little better. They'd be safer, and catch more dinner." ([3], counted p. 29)
**P2**	**Inheritance**		
P2_REP	Reproduction	Does the book mention that living beings reproduce and produce (fertile) offspring?	"Lebewesen sind Dinge, die sich ernähren... die wachsen... sich vermehren." ([12], counted pp.10–12)<br>[Translated: Living beings are things that eat... that grow... reproduce.]
P2_INV	Inherited variation	Does the book mention that the traits/characteristics are inherited from parents to progeny?	"Only the shaggy-furred Borks had survived! So the next time a big Borkling birthing ocurred, all of the babies were born shaggy-furred." ([4], counted pp. 10–11)
**P3**	**Selection**		
P3_RES	Limited resources	Does the video show that natural resources (e.g., space and nutrients) are limited?	"All kinds of living things fought for food and space." ([1], counted p. 17)
P3_SUR	Differences in survival and reproduction rates	Does the book mention that not all individuals of a generation survive to reproduce or have less or more offspring (e.g., due to limited resources or predator attacks)?	"So the next time a big Borkling birthing ocurred, all of the babies were born shaggy-furred." ([4], counted p. 11)
P3_POP	Change in population	Does the book mention that a change in the frequency of characteristics occurs within a population over generations?	"So the next time a big Borkling birthing ocurred, all of the babies were born shaggy-furred." ([4], counted p. 11)

Code	Variable	Operationalization	Example
P3_SPE	Speciation	Does the book mention that new biological species can be formed?	"Weshalb ihnen (...) Füße anstelle von Flossen wuchsen. Und stell dir vor: Jetzt waren sie keine Fische mehr - sie waren Reptilien!" ([7], counted p. 26)<br>[Translated: That is why (...) they grew feet instead of fins. And imagine: Now they were no longer fish - they were reptiles!]
**TC**	**Threshold Concepts**		
**TC1**	**Spatial scales**		
TC1_MOL	Molecule	Does the book mention events at the molecular scale, or in the inherent information that can determine physical aspects and/or can be inherited??	"Neue Arten entstehen durch Mutation. Das ist die Veränderung der Gene. Die Gene enthalten jene Eigenschaften, die uns angeboren sind." ([12], counted pp. 25–26)<br>[Translated: New species are created by mutation. This is the change of genes. The genes contain those characteristics that we are born with.]
TC1_IND	Individual	Does the book mention events at the scale of a single organism without referencing the population or species?	"The dinosaur wanted to fly ... and flying changed the dinosaur into a bird." ([13], counted pp. 9–10)
TC1_POP	Population	Does the book mention that changes occur between organisms of a given group or species in a given geographical area capable of interbreeding?	"So the next time a big Borkling birthing ocurred, all of the babies were born shaggy-furred." ([4], counted p. 11)
TC1_SPE	Species	Does the book mention events beyond the level of one species, i.e., growing cladograms or changes between species?	"Sarkastodons became bears. Hyracotheriums became horses." ([14], counted p. 10)
**TC2**	**Temporal scale**		
TC2_DAY	Seconds / minutes / hours / days	Does the book provide information about temporal changes in seconds, minutes, hours, or days?	"Also bastelte sich der kluge Fisch zwei wunderschöne Paar Füße und zog sie über seine Flossen. Dann, eines schönen Tages, stieg er aus den Wellen und stiefelte den Strand hinauf." ([7], counted pp. 18–20)<br>[Translated: So the clever fish built two beautiful pairs of feet and pulled them over his fins. Then, one fine day, he got out of the waves and strode up the beach.]
TC2_YEA	Years	Does the book provide information about temporal changes in years?	"Once, I was swirling clouds of hydrogen. Over billions of years, I turned into birds, horses, grass, whales." ([15], p. 3)
TC2_GRT	Generations	Does the book indicate time by the progression or counting back of generations?	"Slowly, generation by generation, many of the animals in the ocean change. Some fish even develop real legs." ([16], p. 18)

Code	Variable	Operationalization	Example
TC2_GEO	Geologic timescale	Does the book provide information about temporal changes between epochs, periods, or eras, or use specific terms, e.g., Jurassic or Miocene?	"Vor 251 M.J. : PT-Massenaussterben von 95% aller Arten (Grenze von Perm und Trias)" ([11], p. 21) [Translated: 251 M.Y. ago : PT mass extinction of 95% of all species (boundary of Permian and Triassic)]
TC2_NUM	Time in numbers	Does the book indicate time or years by numbers?	"Over billions of years, I turned into birds, horses, grass, whales." ([15], p. 20)
**TC3**	**Randomness**	Does the book mention the randomness of factors involved in the generation of variation?	"With slight random changes, few were helpful, most, not." ([17], counted p. 9)
**TC4**	**Probability**	Does the book mention the probability of factors affecting individuals' survival or reproduction rate?	"This would make it better at swimming away from attackers and so more likely to survive." ([14], counted p. 5)
**M**	**Misconceptions**		
M1	Transformationism	Does the book present the appearance of new characteristics, changes in populations, or new species without specifying any underlying mechanisms?	"Slowly they evolved into animals more like crabs and fish." ([1], counted p. 18)
M2	Teleology	Does the book present the idea that the evolution of a trait or species was induced by its purpose?	"Und als es kalt wurde, bekamen wir ein Fell. (…) Da wurden wir Ratten und Füchse und Rehe." ([18], counted p. 11) [Translated: And as it got cold, we got fur. (...) Then we became rats and foxes and deer.]
M3	Essentialism	Does the book present species as homogeneous groups that change at the same time and level?	"Aber die Welt war nicht fertig, denn uns wuchsen Knochen. Auf einmal waren wir alle Echsen." ([18], counted p. 7) [Translated: But the world was not finished, because we grew bones. Suddenly we were all lizards.]
M4	Anthropomorphism	Does the book present the idea that phenomena or living beings can make decisions to change?	"Denn eines Tages fassten wir den Entschluss: Wir sind keine Tiere mehr, sagten wir. Denn da hatten wir angefangen zu sprechen. Und dann wurden wir Menschen und wohnten in Höhlen." ([18], counted p. 17) [Translated: Because one day we made the decision: We are no longer animals, we said. Because that was when we had started to talk. And then we became humans and lived in caves.]
M5	Evolution in waves	Does the book present (1) evolution, natural selection, or speciation as a suddenly occurring event or in connection with an event (usually a catastrophe) or (2) the idea that evolution, the appearance of new traits, or speciation start or stop, increase or decrease?	"After an asteroid shattered the calm and a layer of fine dust settled on everything, we changed again." ([19], counted p. 27)

Code	Variable	Operationalization	Example
M6	Recent ancestry	Does the book present recent species as ancestors?	"Der Mensch gehört zu den Primaten. Er stammt von den Affen ab..." ([12], counted pp. 31–32)<br>[Translated: Humans belong to the primates. They descend from apes…]

Note. *„Counted" means that we manually counted the pages of the book as no page numbers were given.

References

1. Barr D, Williams S. The Story of Life: A first book about evolution. London: Francis Lincoln Children's Books; 2015.
2. Morgan J. From lava to life: The universe tells our earth story. 1st ed. Nevada City, Calif.: Dawn Publications; 2003.
3. Reynolds PH, New York Hall of Science. Charlie and Kiwi: An Evolutionary Adventure. 1st ed. New York: Atheneum Books for Young Readers; 2011.
4. Emmett J. How the Borks became: An Adventure in Evolution. Herefordshire: Otter-Barry Books; 2019.
5. Chin J. Island: A Story of the Galápagos. New York: Scholastic Inc.; 2012.
6. Kim EH. Warum hat die Giraffe einen langen Hals?: Wie Tiere sich ihrem Lebensraum anpassen. Frankfurt am Main: Fischer; 2008.
7. Wormell C. Ein kluger Fisch. Weinheim, Basel: Beltz & Gelberg; 2019.
8. Taylor T. Little changes: CreateSpace Independent Publishing Platform; 2012.
9. Bunting P. Ich und der Anfang der Welt. 1st ed. Stuttgart: Gabriel; 2019.
10. Ferrie C, Florance C. Evolution für Babys. 1st ed.: Loewe; 2019.
11. Claybourne A. Faszinierende Evolution: Wie das Leben entstand. Stuttgart: Kosmos; 2019.
12. Eichinger W. Evolution für Kinder. 1st ed. Norderstedt: Qantor Verlag; 2011.
13. Darkin C. How worm became everything: The story of evolution. 1st ed.: Rational Stories; 2019.
14. Layton N. The Story of Everything: From the Bing Bang until now in eleven pop-up spreads: Barron's Educational Series Inc.; 2006.
15. Morgan J. Mammals who morph: The universe tells our evolution story. Nevada City, CA: Dawn Publications; 2015.
16. Bal F, van Doninck S. It started with a big bang: The origin of Earth, you and everything else. Toronto: Kids Can Press; 2018.
17. Becker JR. Annabelle & Aiden in the Story of Life. 1st ed. United States: Imaginarium Press, LLC; 2016.
18. Dahle Nyhus K. Die Welt sagte ja. Berlin: Kullerkupp Kinderbuch Verlag; 2019.
19. Westberg Peters L. Our family tree: An evolution story. 1st ed. San Diego: Harcourt; op. 2003.
