# Supplementary material for: Darwin’s tales–A content analysis of how evolution is presented in children’s books
Source: PLoS One. 2022 Jul 13;17(7):e0269197. doi: 10.1371/journal.pone.0269197 (PMC9278771; doi:10.1371/journal.pone.0269197)
Supplement: S3 Table — (PDF) [file pone.0269197.s003.pdf]

Supporting information

S3 Table

Dictionary of the Computer-Supported Content Analysis

Code	Label	Search terms	Included languages	Additional features	Relative frequency of occurrence (%)		
					in the sample	in NFBs	in SBs
**Evolutionary and biological terms**							
T1	evolution	*evolu*	engl ; ger		45.2	69.2	27.8
T2	trait	trait*<br>characteristic*<br>merkmal*	engl ; ger		16.1	30.8	5.6
T3	ancestry	*ancest*<br>*relat*<br>*vorfahr*<br>*verwandt*	engl ; ger		48.4	53.8	44.4
T4	fossil	*fossil*	engl ; ger		29.0	38.5	22.2
T5	mutation	mutat*<br>mutier*	engl ; ger		12.9	23.1	
T6	other terms	homolog*<br>analog*<br>convergen*<br>konvergen*<br>isolation*<br>atavis*<br>rudiment*	engl ; ger		9.7	23.1	0.0
**Verbs to describe evolutionary change**							
T7	evolve	evolv*	engl		32.3	23.1	38.9
T8	adapt	adapt*<br>an*pass*	engl ; ger		29.0	53.9	11.1
T9	become	bec?m*<br>g?t*<br>*w?rd*	engl ; ger	separate search for English and German terms<br>cases in which the German "werden" is used to build passive voice and future tense (not meaning "to become") were excluded	61.3	69.2	55.6
T10	change	chang*<br>*änder*	engl ; ger		67.7	61.5	72.2

Code	Label	Search terms	Included languages	Additional features	Relative frequency of occurrence (%)		
					in the sample	in NFBs	in SBs
T11	morph	morph*	engl		6.5	0.0	11.1
T12	develop	develop*<br>entwick*	engl ; ger	cases were separated into "use in evolutionary context" and "use without evolutionary context"	32.3	69.2	5.6
T13	appear	emerg*<br>appear*<br>auf*tauch*<br>ersch*n*	engl ; ger		35.5	53.9	22.2
T14	form	*form*<br>*build*<br>*bild*	engl ; ger		22.6	30.8	16.7
T15	grow	gr?w*<br>w?chs*	engl ; ger		35.5	46.2	27.8
**Organismal context**							
T16	bacteria	*bacter*<br>*bakter*	engl ; ger		16.1	23.1	11.1
T17	animals	animal*<br>Tier*	engl ; ger	case-sensitive search	71.0	84.6	61.1
T18	fungi	*fungi*<br>*pilz*	engl ; ger		19.4	30.8	11.1
T19	plants	plant*<br>Plant*<br>Pflanz*	engl ; ger	case-sensitive search	61.3	76.9	50.0
T20	tree	tree*<br>baum*<br>bäume*	engl ; ger		64.5	69.2	61.1
T21	flower	flower*<br>blume*	engl ; ger		12.9	7.7	16.7
**Evolutionary principles**							
T22	variation	variation*<br>variab*	engl ; ger		6.5	7.7	5.6
T23	inheritance	inherit*<br>vererb*	engl ; ger		12.9	23.1	5.6
T24	selection	selec*<br>selek*	engl ; ger		9.7	15.4	5.6

Code	Label	Search terms	Included languages	Additional features	Relative frequency of occurrence (%)		
					in the sample	in NFBs	in SBs
**Threshold concepts**							
T25	dna	dna<br>dns<br>mutier*	engl ; ger		16.1	15.4	16.7
T26	cell	cell*<br>zell*	engl ; ger		32.3	46.2	22.2
T27	population	population*	engl ; ger		12.9	23.1	5.6
T28	species	species<br>Species<br>Art*<br>Spezies	engl ; ger	case-sensitive search	48.4	61.5	38.9
T29	seconds	second*<br>sekunde*	engl ; ger		9.7	0	16.7
T30	minutes	minute*	engl ; ger		3.2	0	5.6
T31	days	day*<br>tag*	engl ; ger		58.1	30.8	77.8
T32	years	year*<br>Jahr*	engl ; ger		80.7	84.6	77.8
T33	large numbers	*hundred*<br>*hundert*<br>*thousand*<br>*tausend*<br>*million*<br>*billion*	engl ; ger		67.7	69.2	66.7

Note. All searches were conducted with the OR logic in MAXQDA, which means that at least one of the terms had to be included in the text. Search results were examined for content and unwanted hits were excluded (for example, "emergency" for the label "emerge"). "?" represents a single character; "*" represents a string of characters.
