# Supplementary material for: Darwin’s tales–A content analysis of how evolution is presented in children’s books
Source: PLoS One. 2022 Jul 13;17(7):e0269197. doi: 10.1371/journal.pone.0269197 (PMC9278771; doi:10.1371/journal.pone.0269197)
Supplement: S4 Table — (PDF) [file pone.0269197.s004.pdf]

Supporting information

S4 Table

Results of the Content Analysis Including Reliability Scores Concerning Organismal Context (O), Principles and Key Concepts (P), Threshold Concepts (TC), and Misconceptions (M)

Code	Variable	Relative frequency of occurrence (%)			Reliability	
		in the sample	in NFBs	in SBs	Krippendorff's alpha	Agreement (%)
O	**Organismal Context**					
**O1**	**Unicellular organisms**					
**O1_UN**	Unspecified	32.3	46.2	22.2	1.0	100
**O1_EU**	Unicellular eukaryotes	6.5	0.0	11.1	1.0	100
**O2**	**Bacteria**	16.1	23.1	11.1	1.0	100
O2_AM	*Number of examples*	Ø 0.0	Ø 0.1	Ø 0.0	1.0	100
O2_S1	*One species*	0.0	0.0	0.0	N/A	100
O2_S2	*Several species*	3.2	7.7	0.0	N/A	100
O2_PL	*Phylogenetic lineage*	16.1	23.1	11.1	1.0	100
O2_RL	*Real species*	3.2	7.7	0.0	N/A	100
O2_RT	*Realistic species*	12.9	15.4	11.1	1.0	100
O2_FK	*Fictitious species*	0.0	0.0	0.0	N/A	100
**O3**	**Animals**	96.8	92.3	100	N/A	100
O3_AM	*Number of examples*	Ø 19.8	Ø 30.2	Ø 12.2	.96	30
O3_S1	*One species*	29.0	0.0	50.0	.81	90
O3_S2	*Several species*	19.4	46.2	0.0	.75	90
O3_PL	*Phylogenetic lineage*	64.5	69.2	61.1	.63	90
O3_RL	*Real species*	74.2	92.3	61.1	.41	80
O3_RT	*Realistic species*	38.7	23.1	50.5	.81	90
O3_FK	*Fictitious species*	12.9	0.0	22.2	1.0	100
**O3_1**	**Humans**	71.0	69.2	72.2	.63	90
**O4**	**Fungi**	9.7	7.7	11.1	1.0	100
O4_AM	*Number of examples*	Ø 0.0	Ø 0.0	Ø 0.0	1.0	100
O4_S1	*One species*	0.0	0.0	0.0	N/A	100
O4_S2	*Several species*	0.0	0.0	0.0	N/A	100
O4_PL	*Phylogenetic lineage*	0.0	0.0	0.0	N/A	100
O4_RL	*Real species*	0.0	0.0	0.0	N/A	100
O4_RT	*Realistic species*	0.0	0.0	0.0	N/A	100

Code	Variable	Relative frequency of occurrence (%)			Reliability	
		in the sample	in NFBs	in SBs	Krippendorff's alpha	Agreement (%)
O4_FK	*Fictitious species*	0.0	0.0	0.0	N/A	100
**O5**	**Plants**	61.3	61.5	61.1	.75	90
O5_AM	*Number of examples*	Ø 1.5	Ø 1.8	Ø 1.3	.94	80
O5_S1	*One species*	0.0	0.0	0.0	0	90
O5_S2	*Several species*	9.7	23.1	0.0	N/A	100
O5_PL	*Phylogenetic lineage*	6.5	7.7	5.6	N/A	100
O5_RL	*Real species*	9.7	15.4	5.6	0	90
O5_RT	*Realistic species*	3.2	7.7	0.0	N/A	100
O5_FK	*Fictitious species*	0.0	0.0	0.0	0	90
**O6**	**Symbols**	3.2	7.7	0.0	N/A	100
**P**	**Principles and Key Concepts**					
**P1**	**Variation**					
P1_ORI	Origin of variation	12.9	15.4	11.1	1.0	100
P1_IND	Individual variation	51.6	46.2	55.6	1.0	100
P1_FIT	Differential fitness	41.9	46.2	38.9	1.0	100
**P2**	**Inheritance**					
P2_REP	Reproduction	54.8	53.8	55.6	.75	90
P2_INV	Inherited variation	48.4	46.2	50.0	1.0	100
**P3**	**Selection**					
P3_RES	Limited resources	35.5	30.8	38.9	1.0	100
P3_SUR	Differences in survival and reproduction rates	54.8	53.8	55.6	1.0	100
P3_POP	Change in population	29.0	30.8	27.8	.81	90
P3_SPE	Speciation	58.1	61.5	55.6	1.0	100
**T**	**Threshold Concepts**					
**T1**	**Spatial scales**					
T1_MOL	Molecule	12.9	23.1	5.6	1.0	100
T1_IND	Individual	9.7	0.0	16.7	.60	80
T1_POP	Population	29.0	30.8	27.8	.04	50
T1_SPE	Species	83.9	92.3	77.8	N/A	100
**T2**	**Temporal scale**					
T2_DAY	Seconds / minutes / hours / days	3.2	0.0	5.6	.41	80
T2_YEA	Years	64.5	69.2	61.1	1.0	100

Code	Variable	Relative frequency of occurrence (%)			Reliability	
		in the sample	in NFBs	in SBs	Krippendorff's alpha	Agreement (%)
T2_GRT	Generations	35.5	38.5	33.3	.81	90
T2_GEO	Geologic timescale	6.5	7.7	5.6	0	90
T2_NUM	Time in numbers	61.3	69.2	55.6	1.0	100
**T3**	**Randomness**	16.1	15.4	16.7	.55	80
**T4**	**Probability**	9.7	15.4	5.6	0	90
**M**	**Misconceptions**					
M1	Transformationism	74.2	84.6	66.7	.75	90
M2	Teleology	51.6	53.8	50.0	.81	90
M3	Essentialism	51.6	69.2	38.9	.42	70
M4	Anthropomorphism	45.2	38.5	50.0	.81	90
M5	Evolution in waves	35.5	38.5	33.3	.42	70
M6	Recent ancestry	16.1	15.4	16.7	.55	80
