# Supplementary material for: Darwin’s tales–A content analysis of how evolution is presented in children’s books
Source: PLoS One. 2022 Jul 13;17(7):e0269197. doi: 10.1371/journal.pone.0269197 (PMC9278771; doi:10.1371/journal.pone.0269197)
Supplement: S1 File — (PDF) [file pone.0269197.s005.pdf]

Supporting Information

S5 Detailed results of the content analysis

Organismal context

Kingdoms and examples

Our findings show that in children's books about evolution, specific contexts and contents tend to be preferred to others. The selected books mainly dealt with animals (97%; *n* = 30; see Fig S5-1). Humans were addressed in 21 (71%), plants in 18 (61%), and fungi in three books (10%). Ten books (32%) mentioned unspecified cells (e.g., "first cells") and two books (7%) referred to unicellular eukaryotes. Five books (16%; three NFBs, two SBs) addressed bacteria but only one specified it by its name. The number of examples ranged between one and 138 animal species and one and eight plant species.

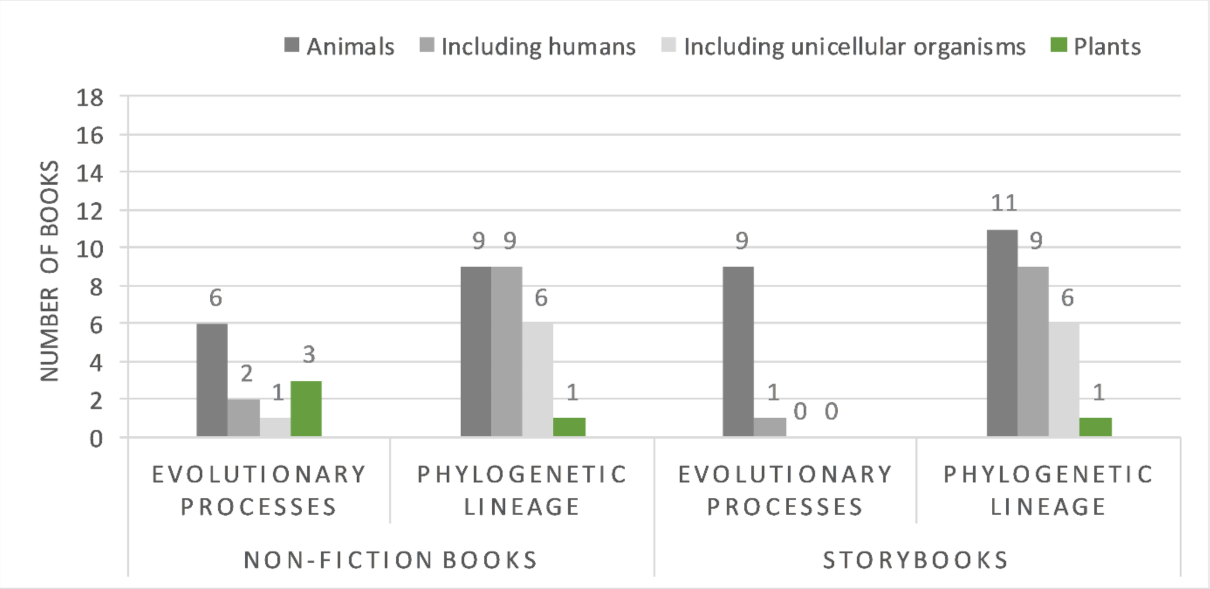

Fig S5-1. Number of books treating evolutionary processes and phylogenetic lineages referring to the animal kingdom (including humans and unicellular animals) and plant kingdom, divided into NFBs (*n* = 13) and SBs (*n* = 18).

None of the books that mentioned fungi (one NFB; two SBs) named a species; they referred to them as "fungi" or "mushrooms". The same occurred for plants in eight books

(three NFBs, five SBs) as they referred to them as "plants", "flowers", "trees", or "forests". The lexical search showed that most of the books contained the labels *animal* (71%), *tree* (65%), and *plant* (61%).

Organismal examples to explain evolution

Most of the examples given in the books, especially those other than animals, were not part of the explanatory sections concerning the topic of evolution. All books that contained animal examples (97%; n = 30) used at least one of them to explain evolution. In contrast, four out of the 18 books (three NFBs; one SB) that contained examples of plants used them to explain evolutionary processes. To explain evolution, the majority of books made use of real examples (n = 23; 12 NFBs and 11 SBs). Realistic examples (in n = 12) mostly consisted of prehistoric species that were reconstructed, but they were either non-specific or were loosely defined by description. Fictitious examples were used in only four SBs in an animal context.

Concerning the evolutionary context, most of the analyzed books (65%) focused on phylogenetic lineages or Earth's history, relying mainly on animal examples. In this context, most books mentioned the evolution of humans (n = 17) and of unicellular organisms (n = 12). Books that covered information concerning evolutionary processes (48%; six NFBs and nine SBs) relied heavily on animal examples and rarely had examples of humans (n = 3), unicellular organisms (n = 1), or plants (n = 3).

Evolutionary Principles and Key Concepts

While most of the analyzed children's books included at least one principle (84%; n = 26; Fig S5-2), they preferred the selection principle to the more basic variation and inheritance principles. All books that covered one (n = 2) or two principles (n = 6; relaxed approach) always included the selection principle (variation and selection, n = 2; inheritance and selection, n = 4; variation and inheritance, n = 0). All three principles were covered in 16 books (52%; relaxed approach). One NFB and one SB addressed all nine key concepts (i.e.,

all principles), while five books did not address any. Using the strict approach (i.e., all key concepts of one principle are present), at least one principle was covered in 12 books (39%).

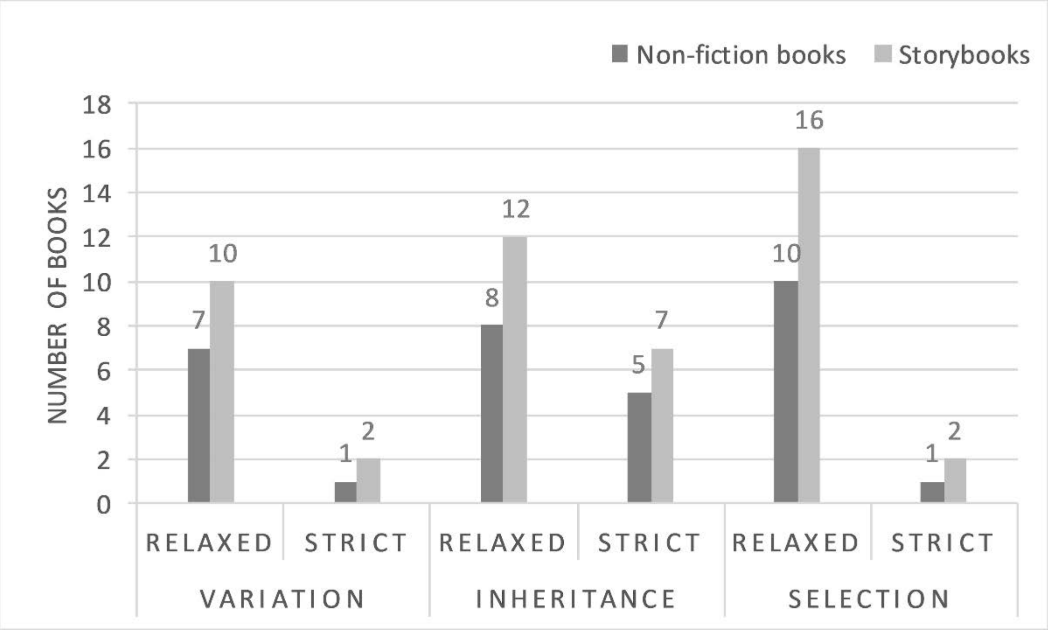

Fig S5-2. Number of books treating the evolutionary principles of variation, inheritance, and selection, calculated with relaxed and strict approaches, divided into NFBs (*n* = 13) and SBs (*n* = 18).

Focusing on the key concepts in particular, more than half of the books addressed the speciation key concept (58%; *n* = 18; NFBs = 8; SBs = 10) and different rates of survival and reproduction (55%; *n* = 17; NFBs = 7; SBs = 10; Fig S5-3), while the least mentioned concept was origin of variation (13%; *n* = 4).

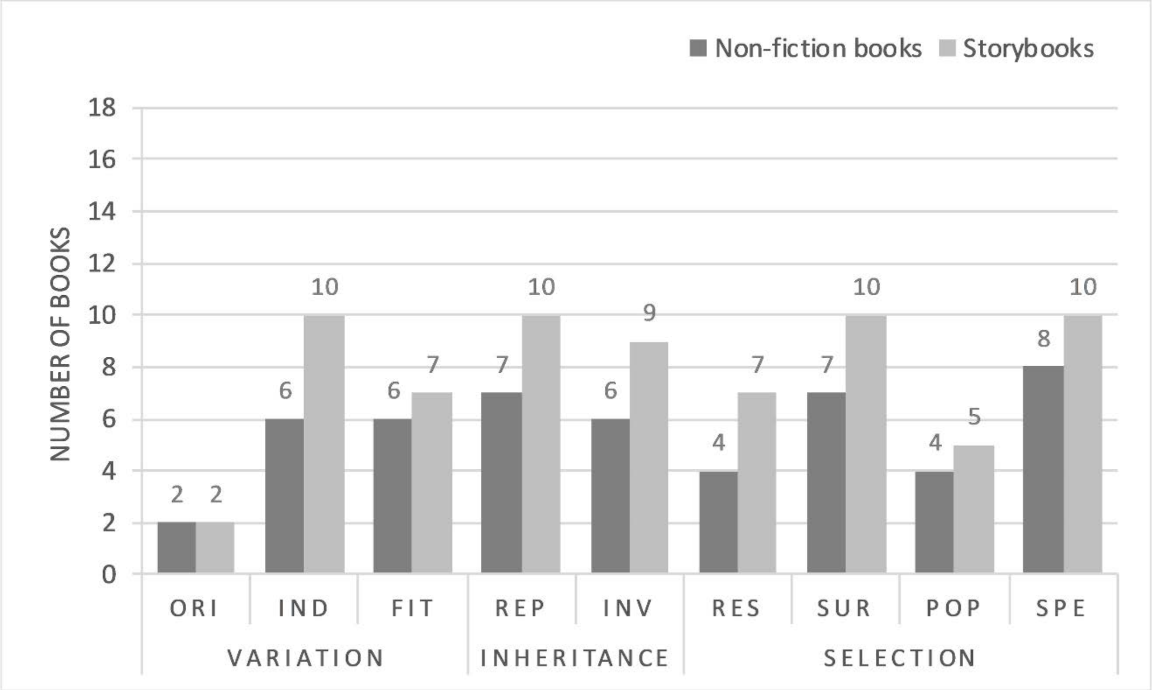

Fig S5-3. Number of books treating the key concepts (Origin of variation [ORI], Individual variation [IND], Differential fitness [FIT], Reproduction [REP], Inherited variation [INV], Limited resources [RES], Different rates of survival and reproduction [SUR], Change in population [POP], and Speciation [SPE]), divided into NFBs (*n* = 13) and SBs (*n* = 18).

Threshold Concepts

Concerning the threshold concepts, the books analyzed favored large spatial and temporal scales while they scarcely considered aspects of randomness and probability. Most books (84%; *n* = 26) treated evolution on the level of species (i.e., speciation or evolution of a species' morphological traits). The term *species* appeared in 15, *cell* in 10, *DNA* in five, and *population* in four books. Moreover, evolution was mostly regarded on a timescale of years (65%) and less often in terms of generations (36%). Changes in short timeframes were largely absent in both NFBs and SBs. The lexical search supported these results, as most of the books used large *numbers* (*n* = 21; i.e., from 100 to several billions of years) and the label *years* (*n* = 25), while the label *days* appeared in 18, *minutes* in one, and *seconds* in three books.

The concept of randomness was mentioned in five books either (a) directly (n = 2, e.g., "slight random changes", Becker, 2016), or (b) with other terms (n = 5; "some were surprises", Emmett, 2019). In three books, we identified the concept of probability from related terms ("probable", "likely"). Again, we did not find differences between NFBs and SBs.

Misconceptions

Overall, we found a high number of misconceptions in the analyzed books (84%, n = 26; Fig S5-4). Most books contained more than three different types of misconceptions. The most prominent reasoning was the use of transformational explanations (74%, n = 23), which also had the highest number of hits per book as it appeared between zero and 51 times. Sixteen books (52%) contained teleological reasoning, and another 16 books (52%) included essentialist ideas. Anthropomorphisms (i.e., conscious decisions to evolve) appeared in 14 books (45%; see Table S5-1 for examples).

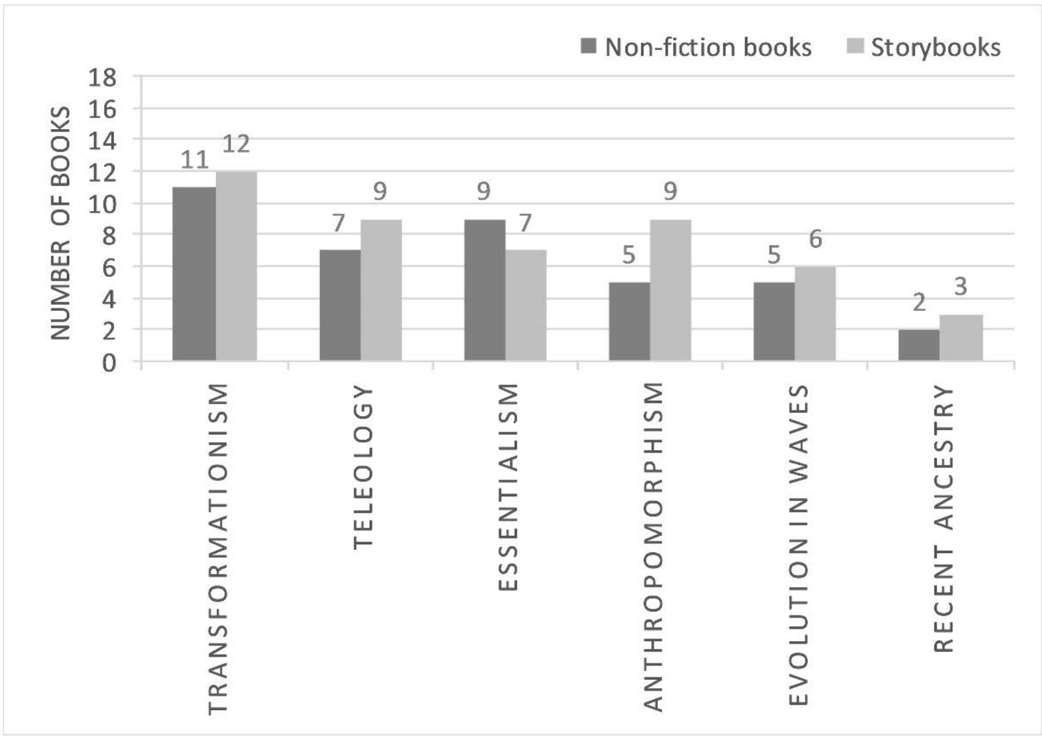

Fig S5-4. Number of books making use of misconceptions, divided into NFBs (*n* = 13) and SBs (*n* = 18).

Table S5-1. Examples of Misconceptions Found in the Books Analyzed

Misconception	Example	Source
Transformationism	"Slowly they evolved into animals more like crabs and fish" (counted* p. 18)	[1]
Teleology	"And then they grew lungs so they could breathe air" (counted p. 9)	[2]
Essentialism	"Over a long time, monkeys evolved into another kind of primate - apes without tails" (p. 16)	[3]
Anthropomorphism	"What mammals chose to return to the sea, where all life once began? Whales and dolphins! " (p. 12)	[3]
Evolution in waves	"In a crisis, I evolve faster." (p. 40)	[3]
Recent ancestry	"Mice can evolve into horses and whales and humans." (p. 40)	[3]

„Counted" means that we manually counted the pages of the book as no page numbers were given.

Lexical Search for Scientific Language

Scientific Terms

We found a higher use of scientific terms in the NFBs. Most of the books (84%) used between one and 12 scientific terms (13 NFBs; 13 SBs). The SBs mentioned scientific terms, on average, six times (minimum: one; maximum: 20) and the NFBs 50 times (minimum: one; maximum: 328). The label *evolution* was used in 69% of the NFBs (n = 9) and 28% of the SBs (n = 5). Terms of the *ancestry* label were used in 48% of the books (n = 15). Other evolutionary terms, such as homology, analogy, or rudiments were found in three NFBs.

Verbs to Describe Evolutionary Change

The occurrence of verbs that describe evolutionary change was high (Fig S5-5). Almost all books (90%; n = 28) used one of the nine labeled verbs (see S3 Table) at least once to describe an evolutionary process. The most frequently used verbs were *change* (n = 21; 68%) and *become/get* (n = 19; 61%). A total of 19 books used at least one verb in another meaning besides the evolutionary context. Most context switches occurred with the verbs *become/get* (n = 16), followed by *change* (n = 10), *grow* (n = 7), and *develop* (n = 6). The verbs *evolve* and *adapt* appeared in 17 books (nine NFBs; eight SBs) and were exclusively used for evolutionary content.

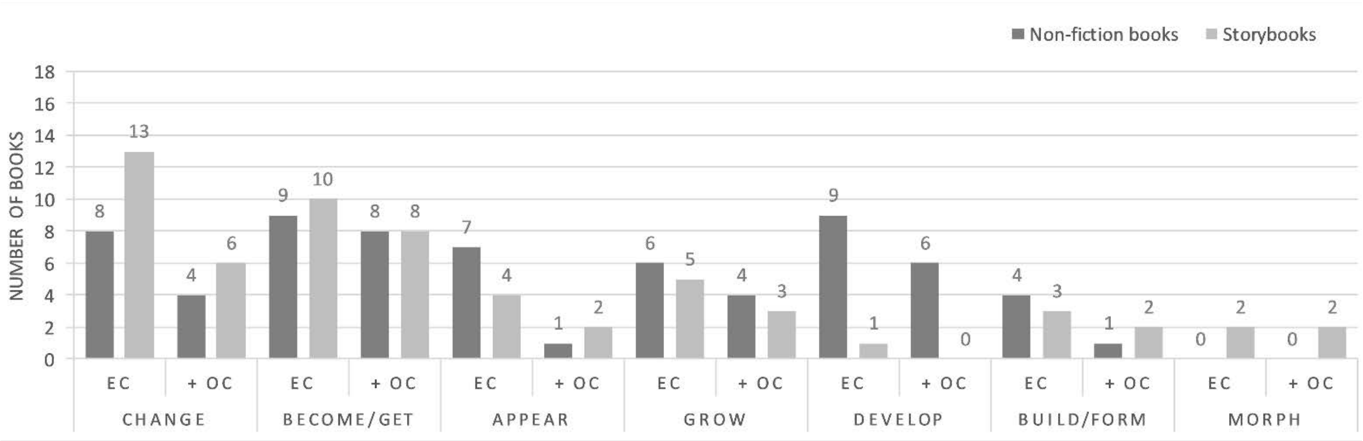

Fig S5-5. Number of books that use verb labels in evolutionary contexts (EC) and also in other contexts (+OC), divided into NFBs (*n* = 13) and SBs (*n* = 18).

Most context switches occurred between evolutionary and geological contexts (28 cases; i.e., times that one verb's meaning switched within one book). Verbs used in an evolutionary context were also used in contexts of conscious decision-making in 15 cases, in a non-biological context in 14, in an ontological context in 11, and in a metabolic context in six cases. Books with an overall low usage (i.e., fewer than two times) of the nine labeled verbs appeared to contain fewer transformational explanations and a lower number of different types of misconceptions.

References

1. Barr D, Williams S. The Story of Life: A first book about evolution. London: Francis Lincoln Children's Books; 2015.
2. Layton N. The Story of Everything: From the Bing Bang until now in eleven pop-up spreads: Barron's Educational Series Inc.; 2006.
3. Morgan J. Mammals who morph: the universe tells our evolution story. Nevada City, CA: Dawn Publications; 2015.
